# A metasurface composed of orifice-type unit cells for the redirection of acoustic waves

**DOI:** 10.1038/s41598-022-18809-1

**Published:** 2022-08-23

**Authors:** Choon Mahn Park, Geo-Su Yim, Sang Hun Lee

**Affiliations:** 1 Department of Materials Physics, Dong-A University, Busan, 49315 South Korea; 2 Department of Electrical Engineering, Pai Chai University, Daejeon, 35345 South Korea; 3 Department of Physics, Sogang University, Seoul, 04107 South Korea

**Keywords:** Acoustics, Mechanical properties

## Abstract

To implement a sound wave redirection system, a two-dimensional (2D) slice of a three-dimensional (3D) metasurface is designed and fabricated using a one-dimensional (1D) face-centred orifice cubic (FCOC) unit cell. The metasurface consists of five identical periodic groups, of which one periodic group consists of eight unit-cell groups with a phase shift of $\pi /4$ adjacent to each other. One unit-cell group consists of four 1D FCOC unit cells with the same orifice diameter. From the numerical simulation results of the designed metasurface, we observed the redirections of sound waves and compared them with the expected theoretical results. It was confirmed that the experimental results agree well with the simulated results with respect to the different incident angles and frequencies. The used frequencies that satisfy the homogeneous medium condition of the metamaterial for the redirection of incident waves range between 1500 and 2700 Hz. At the characteristic frequency of 1540 Hz at normal incidence, it is considered that stationary evanescent waves exist at the boundary of the metasurface due to the characteristics of the surface wave and the limited end boundary. The FCOC-based metasurface provides a new method of metasurface fabrication and is expected to expand the applicability of the metasurface because it can be easily applied to a surface with any shape.

## Introduction

Since the concept of metamaterials was introduced in 1967 by Veselago, in-depth and varied studies on metamaterials for electromagnetic waves and acoustic waves, respectively, have been conducted^1–4^. In particular, due to the possibility of implementation in real life, research on metamaterials having a negative refractive index that cannot be seen in natural products and their applications is being actively conducted. As a result, it was found that it was not impossible to create an invisibility cloak and/or stealth object with a hiding function that was only possible in the virtual world by changing the shape of the structure of materials^3,4^. In this case, due to the structural shape and size of the artificial material, we can observe complex phenomena that we have not been able to understand until now^5–8^.

When a wave is incident on a uniform and normal material, the well-known Snell’s law can be used if the plane of reflection or transmission of the wave can be regarded as a structurally uniform plane. If the plane of material for the incident waves has structurally periodic or some spatial characteristics with respect to the wavelength, the ’generalized Snell’s law’, including the term of spatial characteristics of the plane, should be used for the wave propagation^9,10^. Therefore, using the generalized Snell’s law and an artificially made metamaterial with finite thickness (or metasurface), the direction of reflected and/or transmitted waves can be changed by the artificial structural properties of the metamaterial for the incident wave^11,12^. If we can artificially change the direction of reflection or transmission of a wave in this way, we can hide the predicted location of the object or pretend to be in the wrong location^13^. By doing so, we can give objects stealth capabilities or implement a more effective invisibility cloak. In particular, to realize the stealth characteristics of sound waves, a metasurface composed of unit cells with various structures, such as labyrinthine-type corridors and Helmholtz resonators, was studied. Multiple periodic groups of unit cells made up of Helmholtz resonators or labyrinthine corridors can be used to create metasurfaces that change the direction of reflected or transmitted waves. Helmholtz resonators with different resonant frequencies for the incident waves can give different effective refractive indices due to different wave velocities^11,14,15^. A labyrinthine corridor with different path lengths for the wave can provide an effective phase difference for the incident waves^16–18^. In this way, the aforementioned stealth function for sound waves can be implemented by using the characteristics of waves that emit with different phases with respect to the incident wave. A 1D FCOC unit cell is a cubic orifice-type unit cell that can achieve different effective refractive index values by varying the structure ratio between the orifice diameter and the unit cell length, where the structure ratio is defined as the ratio of the orifice diameter to the length of the unit cell. Using this property, we succeeded in realizing a convex lens, a GRIN lens, and a Luneberg lens for acoustic waves^19–21^. In addition, we developed a hemispherical acoustic Luneberg lens with the same characteristics but different shapes than ordinary spherical Luneburg lenses by applying a conformal transformation for sound waves^22^.

In this paper, we designed a metasurface on which several groups of 1D FCOC metamaterial unit cells with different structure ratios are arranged periodically, and in so doing, the value of the effective refractive index changes periodically. It is found through computational simulations that the direction of the incident sound wave can be changed depending on the structural characteristics of the designed metasurface in this way, and this is certified through experiments. Thus, it is confirmed that the 1D FCOC unit cell can be used to implement an acoustic metasurface that can provide a stealth function for sound waves.

### Design and fabrication of the experimental setup

**Figure 1:**
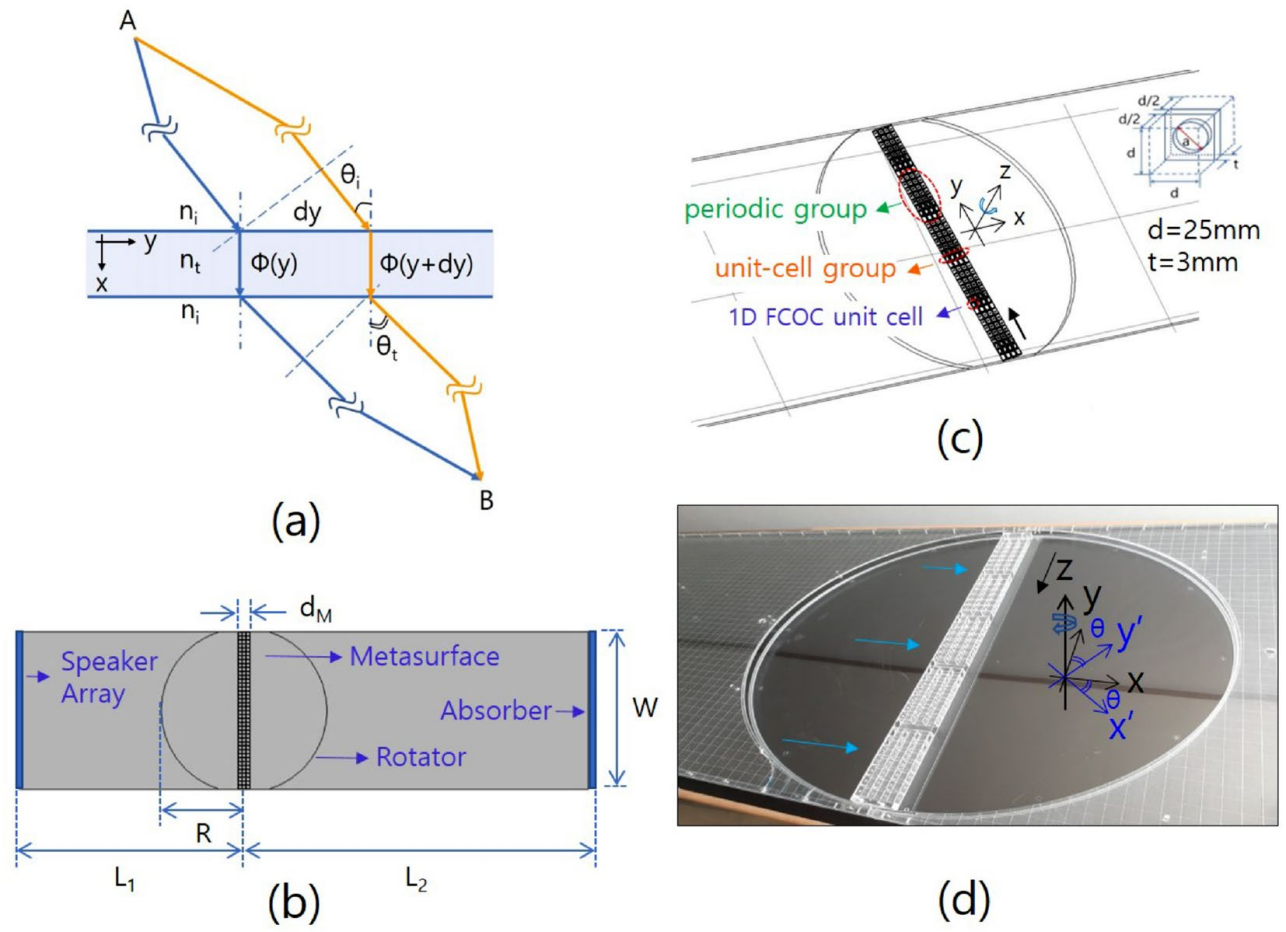
(**a**) shows the schematic diagram for the phase change of the wave refraction due to the metasurface with different refractive index values with respect to the incident point of the surface. (**b**) shows the experimental setup: this experimental setup consists of a speaker array for the sound source, a metasurface boarded on rotatable circular plates (or rotator), and an absorber located on the right-hand end boundary. The lengths of $\mathrm {L_1}$, $\mathrm {L_2}$, W, R and $\mathrm {d_M}$ are 1590, 1775, 1150, 575 and 87 mm, respectively. The height of the waveguide is 25 mm. (**c**) shows the designed metasurfaces composed of the 1D FCOC unit cells. The inset in the figure represents the 1D FCOC unit cell. The volume of the cavity of the unit cell is 15.625 cm$^3$ with a length of d = 25 mm and thickness of t = 3 mm. (**d**) shows the fabricated metasurface, where the top cover plate of the rotator and the microphone have been removed for a clear image of the metasurface. The image of the thick line running through the center of the figure is a mirror image of the edge of the laboratory ceiling. The cyan arrows indicate incident sound waves.

As can be seen from the schematic diagram in Fig. 1a, when the wave propagation paths are different, different phase changes of wave refraction occur due to metasurfaces with different refractive index values for the point of incidence on the surface. We designed a metasurface with a distribution of refractive index values to realize a wave that is refracted in different directions with respect to the direction of incidence of the wave. The fabricated experimental setup is shown in Fig. 1b. This experimental setup consists of a speaker array for the sound source, a metasurface boarded on rotatable circular plates, and an absorber located on the right-hand end boundary. Figure 1c, d show the designed and fabricated metasurface composed of the 1D FCOC unit cells, where the length of the 1D FCOC unit cell is 28 mm. The inset of Fig. 1c shows the 1D FCOC unit cell. The volume of the cavity of the unit cell is 15.625 cm$^3$, and the orifice on the wall of the unit cell is created by drilling a hole in the centre of a 3.0-mm-thick acrylic plate with a diameter that is calculated using Eq. (1).

The effective refractive index $n_{eff}$ of the 1D FCOC unit cell can be derived as follows^19^:

1 $$\begin{aligned} n_{eff} = n_{o} \left[ 1 + \left( t' / d \right) (S_w- S_{or})/S_{or} \right] ^{1/2}. \end{aligned}$$

In Eq. (1), $n_0$ is the refractive index of air, $t'$ is the effective thickness, *d* is the unit cell length, and $S_w$ and $S_{or}$ are the cross-sectional area of the waveguide and that of the orifice, respectively. The metasurface consists of five identical periodic groups, where one periodic group consists of eight unit-cell groups. Each unit-cell group has a phase change of $\pi /4$ between the adjacent unit-cell groups. In the design of the metasurface, the working frequency is 2000 Hz with a sound velocity of 345 m/s. A unit-cell group consists of four 1D FCOC unit cells with the same orifice diameter. The lengths of the periodic group and the metasurface are 224 and 1123 mm, respectively. The thickness of the metasurface $\mathrm {d_M}$ is 87 mm ($\simeq 0.5 \lambda$). To redirect the incident plane waves with respect to the different incidence angles, the metasurface is mounted between two circular plates, as shown in Fig. 1c. These circular plates with the onboarded metasurface (or rotator) are inserted between the hollowed incident and transmitted regions with the same size in the 2D waveguide to make the rotation possible. The diameter of the rotator is approximately 1150 mm. The black arrow on the metasurface indicates the decrement direction of the orifice diameter of the unit-cell group. As in Fig. 1a, the phase difference of acoustic waves propagating through the metasurface, which has a periodic distribution of refractive index, satisfies the following equation^10,16,23^:

2 $$\begin{aligned} \left[ k_0 \; n_i \; \mathrm{{sin}}\; \theta _t \; dy + \phi \right] - \left[ k_0 \; n_i \; \mathrm{{sin}} \; \theta _i \; dy + (\phi + \Delta \phi )\right] = 2\pi \; m. \end{aligned}$$

$\theta _i,\;\theta _t, \;k_0, \;n_i,$ and $n_t$ are the incident angle, transmitted angle, wavenumber in the air, refractive index of the air, and that of the metasurface, respectively, where *m* is a positive or negative integer including zero. From Eq. (2), the transmitted angle $\theta _t$ can be derived as follows:

3 $$\theta_t = {\rm sin^{-1}} \left({\rm sin} \theta_i + \frac{\nu_c}{\nu} m \right), \;{\nu_c} = \frac{c \xi}{2\pi}, \; \xi = \frac{d\phi}{dy}.$$

The characteristic frequency $\nu _c$ is defined as $c \xi /2 \pi$ for the consideration of the evanescent waves, where $\xi$ is the phase gradient of the metasurface and is approximately 8.93$\pi$ rad/m. The value of $\nu _c$ can be calculated as 1540 Hz.

**Figure 2:**
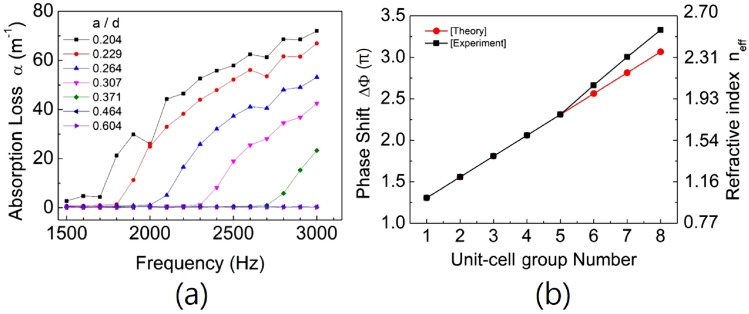
(**a**) shows the values of the absorption loss for the acoustic waves with respect to the frequency and structure ratio of the unit-cell group of the metasurface. (**b**) shows the change in the phase shift of the unit-cell group that consists of four 1D FCOC unit cells. In this figure, the unit-cell group number indicates the order of the unit-cell group in the periodic group. These adjacent unit-cell groups are designed to have different phase values of $\pi /4$ to each other. Black squares and red dots with lines show the experimental and theoretical values of the phase change (or refractive index) of the unit-cell groups with different structure ratios.

**Figure 3:**
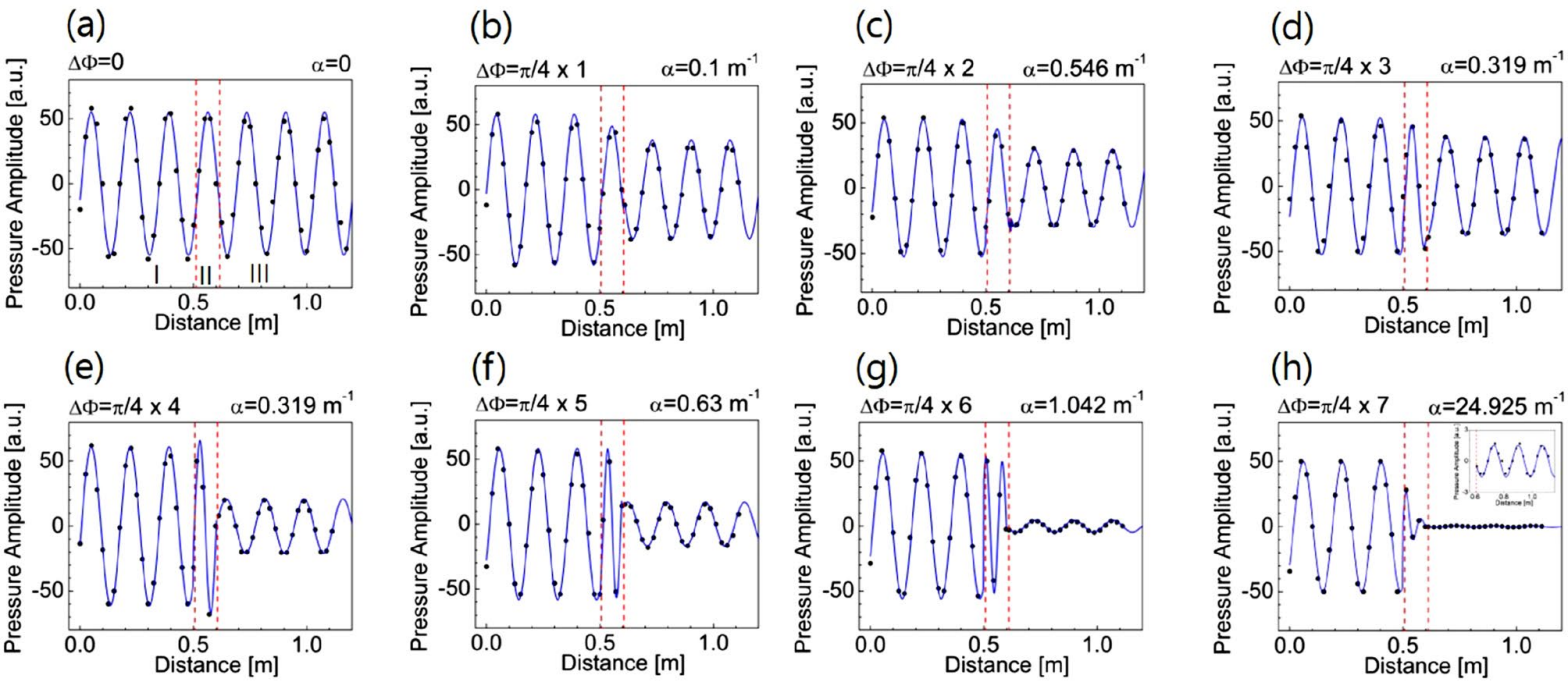
Sound propagation through the unit-cell groups with the same phase change of $\pi /4$ between the unit-cell groups adjacent to each other at the reference frequency. Black dots represent the measured values from the experiment, and the blue solid lines indicate their numerical fit results. In each graph, a unit-cell group consisting of four 1D FCOC unit cells with the same orifice diameter is located between the normal waveguides (in the red dotted line). The inset in figure (**h**) shows a 3$\times$ image of the transmitted sound amplitude.

Figure 2a shows the absorption loss $\alpha$ for the acoustic waves with respect to the frequency and structure ratio (indicated by marked symbols) of the unit-cell group of the metasurface. Figure 3 shows the sound propagations through the eight unit-cell groups at the working frequency of 2000 Hz. Black dots represent the measured values from the experiment, and the blue solid lines indicate their numerical fit results. From the results of Figs. 2a and 3, the values of $n_{eff}$ for different orifice-type unit-cell groups can be determined experimentally. In each graph of Fig. 3, a unit-cell group consisting of four 1D FCOC unit cells with the same orifice diameter is located between the normal waveguides (in the place of the red dotted line). The higher the order of the unit-cell group in the periodic group, the greater is the absorption loss of the transmitted sound. The inset in Fig. 3h shows a 3X image of the transmitted sound amplitude. When the value of the structure ratio *a*/*d* is the same or greater than the value of 0.307 in Fig. 2a, the measured values of $n_{eff}$ are nearly equal to the theoretical values (from (a) to (e)). When the value of the structure ratio is smaller than 0.307, the measured values of $n_{eff}$ are greater than the theoretical values (from (f) to (h)). The experimental values of $n_{eff}$ (from (f) to (h)) increase approximately 3.95, 6.73 and 7.94% with respect to those theoretical values, respectively. The results are shown in Fig. 2b, where the black squares and red dots with lines show the experimental and theoretical values of the phase change (or the corresponding effective refractive index) of the unit-cell groups with different structure ratios, respectively. This result shows the acoustic nonlinear effect having the threshold for the 1D FCOC unit cell. The threshold is at the 5th unit-cell group with a structure ratio value of 0.307 in the periodic group.

## Results and discussion

**Figure 4:**
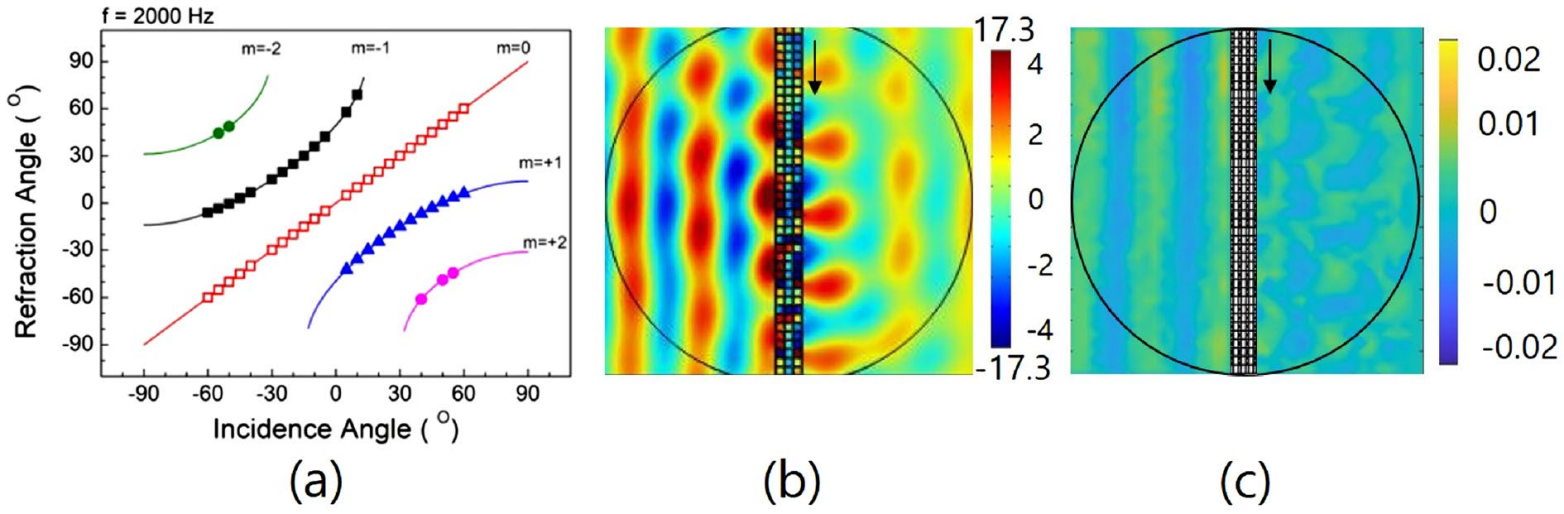
(**a**) shows the relation between the incident angle and the refractive angle of sound waves by the designed metasurface. The solid lines and marked symbols are the theoretically calculated and numerically simulated results, respectively. The reference frequency and the phase gradient of the designed metasurface are 2000 Hz and 8.93$\pi$ rad/m, respectively. (**b**) shows the simulation results at the characteristic frequency of 1540 Hz at normal incidence, and (**c**) shows the experimental results.

The relations between the incident and refractive angles of sound waves created by the designed metasurface are shown in Fig. 4a. The solid lines and marked symbols indicate the theoretically calculated and numerically simulated results, respectively. *m* is a diffraction order by the periodic groups of the metasurface. When the sound waves are normally incident to the metasurface, it is possible that there are three modes for sound waves to propagate through the metasurface (*m* = 0, + 1, − 1). The waves of mode *m* = 0 show propagation through the metasurface without refraction. The waves of modes *m* = − 1 and + 1 show the propagations of waves after the redirections due to the phase changes from the metasurface. It is well known that when the incident angle is equal to the critical angle $\theta _c$ in the total internal reflection, sound waves cannot transmit, and only evanescent waves exist at the boundary. In this case, the evanescent waves can take the form of a standing wave due to the finite bounded length of the metasurface in the transmitted region. At the characteristic frequency $\nu = \nu _c$ at normal incidence, with the help of Eq. (3), the effect of much reflection on the incident surface and the stationary evanescent waves formed on the transmitted side of the metasurface are observed in Fig. 4b^24,25^. Figure 4c shows the experimental result at the characteristic frequency of 1540 Hz. From the results in Fig. 4b, c, we can observe that there are very weak pressure amplitudes after passing through the metasurface, and approximately five agglomerates corresponding to five wavelengths are formed on the boundary of the metasurface in the transmitted region. This is because the wavelength of the characteristic frequency is equal to the value of the length of one periodic group of the designed metasurface, and there are five periodic groups in the metasurface. Using a numerical simulation, the characteristic frequency is actually determined to be 1560 Hz, which is approximately 1.3% larger than the theoretically calculated value of 1540 Hz. This 1.3% increase in the characteristic frequency in the numerical analysis appears to be due to the nonlinear increase in the refractive index with respect to the unit-cell group number (or structure ratio), as shown in Fig. 2b. This increase in the effective refractive index contributes to an increase in the amount of phase change and an increase in the characteristic frequency of the metasurface.

**Figure 5:**
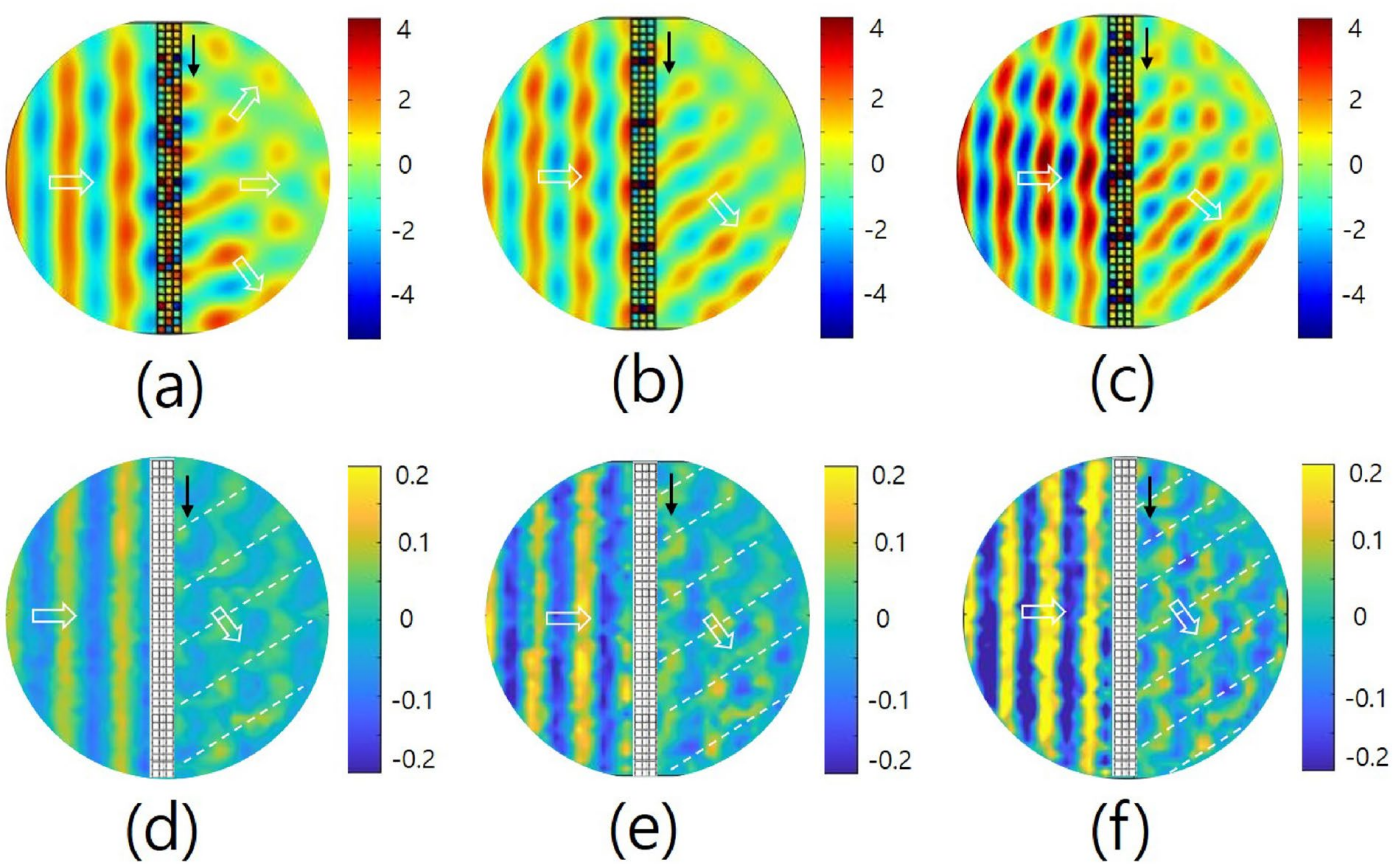
The propagation of sound waves after passing through the metasurface with respect to the frequencies at normal incidence. (**a**–**c**) are the results of the numerical simulation, and (**d**–**f**) are the corresponding results of the experimental measurement. The white arrows are the propagation directions of sound waves incident and after transmission, and the black arrow near the metasurface indicates the increment direction of the effective refractive index of the metasurface. The driving frequencies of figures (**a**)–(**c**) are 1700, 2000 and 2300 Hz, respectively. The experimental (theoretical) values of the refractive angles are approximately $-\,55^{\circ }(-\,65.0^{\circ }), -\,48^{\circ }(-\,50.4^{\circ })$ and $-\,46^{\circ }(-\,42.0^{\circ })$, where we set the sign of the angle to be positive when the angle is counterclockwise from the normal.

Figure 5 shows the propagation of sound waves after passing through the metasurface with respect to the frequencies at normal incidence. The driving frequencies of Fig. 5a–c are 1700, 2000 and 2300 Hz, respectively. These figures are the results of the numerical simulation, and Fig. 5d–f show the corresponding results of the experimental measurement. The experimental (theoretical) values of the transmitted angles are approximately $-\,55^{\circ }(-\,65.0^{\circ }), -\,48^{\circ } (-\,50.4^{\circ })$ and $-\,46^{\circ } (-\,42.0^{\circ })$. In the figures, we set the sign of the angle to be positive when the angle is counterclockwise from the normal. As expected from the result in Fig. 4a, there are three possible modes (*m* = – 1, 0, + 1) at normal incidence for the propagation direction of the wave after passing through the metasurface. The white arrows indicate the propagation directions of sound waves incident and after transmission, and the black arrow near the metasurface indicates the increment direction of the effective refractive index of the unit-cell group of the metasurface. At frequencies greater than or equal to the designed working frequency of 2000 Hz, the refraction angle of the wave is nearly equal to the theoretical value. However, it is far from the theoretical value at a frequency of 1700 Hz. This can be interpreted as follows: at a frequency smaller than the reference frequency, the *m* = 0 mode dominates over the *m* = + 1 mode, and at a frequency greater than the reference frequency, the *m* = + 1 mode dominates over the *m* = 0 mode. The reason that the mode of *m* = + 1 is more dominant than the mode of *m* = – 1 is because the refractive index distribution of the metasurface has directionality, and the transmittance of the wave depends on the position of the metasurface^10,24^. In the optical devices, a blazed grating which is a special type of diffraction grating has the characteristic of making the diffraction distribution asymmetric with respect to the incident wave due to the blazed shape^26^. Since the metasurface composed of periodic groups has a directional distribution of refractive index similar to that of the blazed grating, it seems to have a similar effect to the blazed grating. The wave propagation at normal incidence seems more complicated in the experiment than in the simulation because there are three possible modes at normal incidence and the influence of the m = 0 mode is not small compared to the other two modes (m = + 1, – 1) in the experiment.

**Figure 6:**
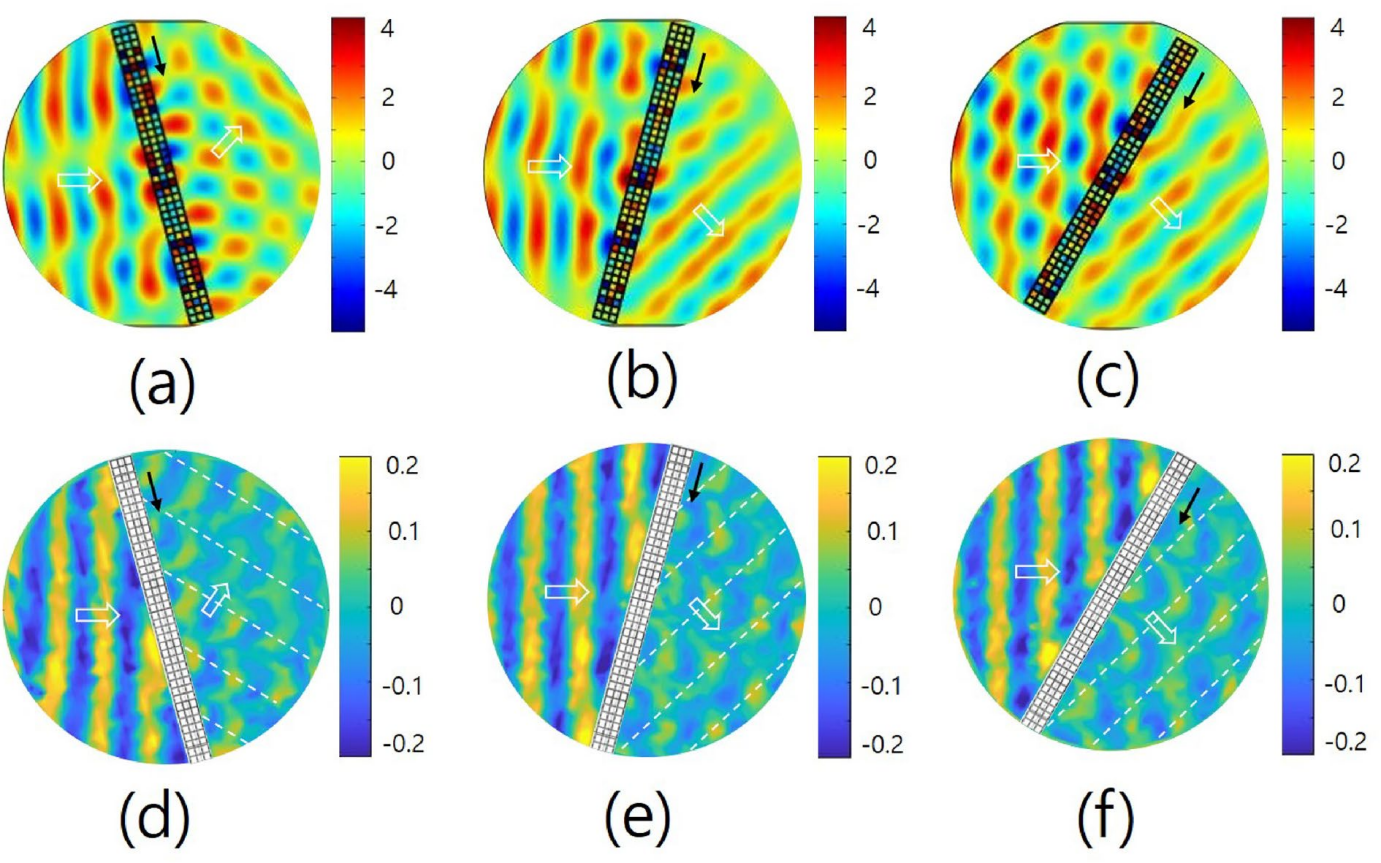
Propagations of sound waves after passing through the metasurface with respect to the incident angles for the metasurface at the characteristic frequency of 2000 Hz. (**a**–**c**) are the results of the numerical simulation, and (**d**–**f**) are the corresponding results of the experimental measurement. The incident angles of sound waves of (**a**)–(**c**) are $-\,15^{\circ }, +\,15^{\circ }$ and $+\,30^{\circ }$, respectively. The experimental (theoretical) values of refractive angles are determined as $40^{\circ } (30.8^{\circ }), -\,33^{\circ } (-\,30.8^{\circ })$ and $-\,15^{\circ } (-\,15.7^{\circ })$, respectively.

Figure 6 shows the propagation of sound waves after passing through the metasurface with respect to the incident angles for the metasurface at a frequency of 2000 Hz. Figure 6a–c show the results of the numerical simulation, and Fig. 6d–f show the corresponding results of the experimental measurement. In Fig. 6a–c, the incident angles of sound waves are $-15^{\circ }, +15^{\circ }$ and $+30^{\circ }$, respectively. In the figures, the redirections of waves after passing through the metasurface are clearly observed. The experimental (theoretical) values of the refractive angles are determined as $40^{\circ } (30.8^{\circ }), -\,33^{\circ } (-\,30.8^{\circ })$ and $-\,15^{\circ } (-\,15.7^{\circ })$, respectively, in Fig. 6d–f. The diffraction orders are *m* = – 1 for the case of (a) and + 1 for the cases of (b) and (c). In Fig. 6a compared to Fig. 6b, it can be seen that the propagating wave is more locally agglomerated than a plane wave after passing through the metasurface. This is considered to be due to the different directions of the phase gradients of the periodic group of the metasurface.

## Experiment and methods

A 21-speaker array with a length of 1150 mm is located at the left-hand end boundary of the waveguide, as shown in Fig. 1b, to generate acoustic plane waves. The acoustic plane waves generated by the sound sources enter the metasurface with the incidence angle. To change the direction of incidence to the metasurface, we rotate the device counterclockwise (or clockwise) for the negative (or positive) angle of incidence. Because of the frequency constraint of the homogeneous medium condition of the metamaterial (i.e., $d \le \lambda /4$) and the attenuation losses of the system for various frequencies, the highest and lowest frequencies for the employed metamaterial are approximately 1500 Hz ($d \simeq \lambda /8$) and 2700 Hz ($d \simeq \lambda /5$), respectively^8^. The measured value of the sound velocity is 344 ± 1.0 m/s in the waveguide. Amplitude-shift-keying modulated acoustic pulses with a width of 3 ms and frequency of 2.75 Hz are used to eliminate the echoes stemming from the multiple reflections that occur at the boundaries of the waveguide^27^. These acoustic pulses are activated simultaneously and in phase using a functional generator. Since the maximum value of the refractive index of the metasurface is approximately 2.55, we set the sample time for the measurement to 4 ms in the incident region and 7.65 ms in the transmitted region to consider the elongation effect of the transmitted wave. A sound absorber is used on the right-hand end of the waveguide to minimize any reflected waves and is experimentally confirmed to operate in our required frequency range. To obtain a change in the direction of the wave propagation, the pressure amplitudes and the retarded times from the sound source are measured at a one unit-cell-length interval along the rotated x and y directions in the circle plates equipped with the metasurface using a condenser-type microphone. To increase the reliability of the measurement, the root-mean-squared value of the pressure amplitude and the retarded time for the sound wave at the measured position are obtained simultaneously by measuring the sound wave signals five times at each position. We also use a low-pass filter with a cut-off frequency of 5 kHz on the measurement system to remove the noise signal.

## Conclusion

We designed and fabricated a metasurface to redirect the incident acoustic waves. After numerical simulation, we fabricated an experimental setup of a 2D slice of a 3D metasurface using 1D FCOC unit cells. This metasurface consists of five identical periodic groups, and one periodic group consists of eight unit-cell groups adjacent to each other with a phase shift of $\pi /4$. One unit-cell group consists of four 1D FCOC unit cells with the same orifice diameter. From the results of experiments along with numerical simulations, we have successfully observed and confirmed the direction change of incident acoustic plane waves by the metasurface with respect to the different incident angles and frequencies. In addition, at the characteristic frequency at normal incidence, it is considered that localized sound waves exist at the boundary of the metasurface in the transmitted region due to the characteristics of the evanescent wave and the limited end boundary.

## Data Availability

The datasets used and/or analysed during the current study available from the corresponding author on reasonable request.
